# Measurement of skeletal related events in SEER-Medicare: a comparison of claims-based methods

**DOI:** 10.1186/s12874-015-0047-5

**Published:** 2015-08-19

**Authors:** Abdalla Aly, Eberechukwu Onukwugha, Corinne Woods, C. Daniel Mullins, Young Kwok, Yi Qian, Jorge Arellano, Arun Balakumaran, Arif Hussain

**Affiliations:** Department of Pharmaceutical Health Services Research, University of Maryland, School of Pharmacy, Saratoga Building, 12th Floor, 220 Arch Street, Baltimore, MD 21201 USA; Pharmaceutical Research Computing, University of Maryland, School of Pharmacy, Saratoga Building, 12th Floor, 220 Arch Street, Baltimore, MD 21201 USA; University of Maryland Medical Center, 22 S. Greene Street, Baltimore, MD 21201 USA; Amgen Inc., 1 Amgen Center Drive, Thousand Oaks, CA 91320 USA

**Keywords:** Skeletal related events, Administrative claims, SEER-Medicare, Measurement, Metastatic prostate cancer

## Abstract

**Background:**

Skeletal related events (SREs) are common in men with metastatic prostate cancer (mPC). Various methods have been used to identify SREs from claims data. The objective of this study was to provide a framework for measuring SREs from claims and compare SRE prevalence and cumulative incidence estimates based on alternative approaches in men with mPC.

**Methods:**

Several claims-based approaches for identifying SREs were developed and applied to data for men aged [greater than or equal to] 66 years newly diagnosed with mPC between 2000 and 2009 in the SEER-Medicare datasets and followed through 2010 or until censoring. Post-diagnosis SREs were identified using claims that indicated spinal cord compression (SCC), pathologic fracture (PF), surgery to bone (BS), or radiation (suggestive of bone palliative radiation, RAD). To measure SRE prevalence, two SRE definitions were created: ‘base case’ (most commonly used in the literature) and ‘alternative’ in which different claims were used to identify each type of SRE. To measure cumulative incidence, we used the ‘base case’ definition and applied three periods in which claims were clustered to episodes: 14-, 21-, and 28-day windows.

**Results:**

Among 8997 mPC patients, 46 % experienced an SRE according to the ‘base case’ definition and 43 % patients experienced an SRE according to the ‘alternative’ definition. Varying the code definition from ‘base case’ to ‘alternative’ resulted in an 8 % increase in the overall SRE prevalence. Using the 21-day window, a total of 12,930 SRE episodes were observed during follow up. Varying the window length from 21 to 28 days resulted in an 8 % decrease in SRE cumulative incidence (RAD: 10 %, PF: 8 %, SCC: 6 %, BS: 0.2 %).

**Conclusions:**

SRE prevalence was affected by the codes used, with PF being most impacted. The overall SRE cumulative incidence was affected by the window length used, with RAD being most affected. These results underscore the importance of the baseline definitions used to study claims data when attempting to understand relevant clinical events such as SREs in the real world setting.

## Background

The skeleton is the most common site of metastasis in patients with advanced solid tumors [1, 2]. Approximately 65–75 % of patients with advanced prostate or breast cancer develop bone metastasis during the course of their disease [3]. Randomized clinical trials (RCTs) have demonstrated that malignant bone lesions resulting from metastatic cancer have significant clinical consequences that contribute substantially to the burden of disease and reduce quality of life [4–7]. These clinical consequences, known as bone complications, also commonly referred to as skeletal related events (SREs), include pathologic fracture (PF), spinal cord compression (SCC), bone palliative radiotherapy (RAD), and bone surgery (BS).

The Food and Drug Administration (FDA) has designated SREs as a measurable composite endpoint allowing investigators to evaluate efficacy of drugs to prevent SREs [8]. Zoledronic acid and, more recently, denosumab have been approved by the FDA for the prevention of SREs based on delaying their onset [9]. When measuring SREs in RCTs to assess clinical burden, a 21-day window for counting events was imposed whereby any SRE occurring within 21 days of a previous one was not counted as a new SRE. This approach is clinically meaningful in the context of a RCT for establishing efficacy because individual SREs may be serially interdependent. For example, a bone surgery performed to treat a spinal cord compression event that occurred within 21 days of the bone surgery is considered a single SRE.

**Fig. 1:**
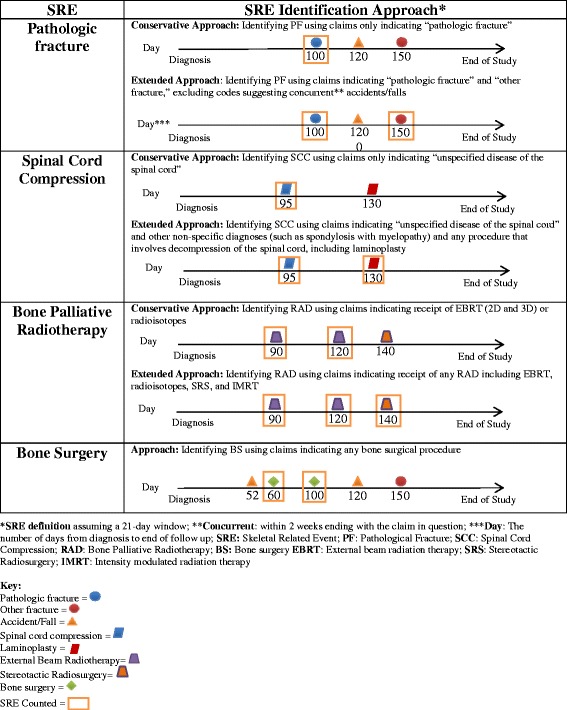
SRE identification approaches from claims

Studies using administrative claims data have also demonstrated that patients with bone metastasis and SREs have high utilization of healthcare resources and high associated costs [10–16]. Contrary to RCTs, where measuring SREs is supported by radiologic evaluations and frequent patient follow up in a prospective fashion, it is less clear how investigators can accurately identify SREs from claims data. There are multiple ways in which investigators have identified SREs using administrative claims, ranging from approaches that maximized sensitivity of SRE measures to methods that aimed at higher specificity for specific SRE subtypes [11–19] (Fig. 1). Moreover, the potential for misclassification may differ across subcomponents of SREs when using claims data to identify the occurrence of an SRE. The accurate identification of an SRE depends on a reliable approach for identifying both the occurrence of SRE subcomponents and their sequencing. The 21-day window for grouping SRE subcomponents is accepted from a regulatory standpoint by the FDA and applied in RCTs [20–22]. It is not clear whether the 21-day window used in RCTs is appropriate for identifying unique SREs when using claims data.

Given the variability in the methods for studying SREs using claims data, we undertook the present study in an effort to better understand and clarify the impact of the various approaches used in the identification of SREs from administrative claim datasets. In particular, in this paper we focus on the measurement of SRE prevalence and cumulative incidence, both of which are commonly measured in administrative claims data to answer clinical and economic questions. We address SRE measurement issues by analyzing two relevant questions. First, *what are the most appropriate and relevant claims to use to identify SREs?* Second, *given the claims used, what is the time period during which claims should be clustered to represent one SRE episode?* The potential for overestimation or underestimation of SRE measures is a function of the claims used and/or the time period used to cluster the claims. For cumulative incidence, we adopted the RCT 21-day window as the base case and provide alternative measures by varying the window length. The goal of this study is to provide a framework for measuring SREs from claims and compare SRE prevalence and cumulative incidence estimates based on alternative measurement approaches using metastatic prostate cancer (mPC) as a case study.

## Methods

### Data sources and sample selection

We estimated and compared prevalence and cumulative incidence of SREs using the Surveillance, Epidemiology, and End Results (SEER)—Medicare linked datasets. This database links information from the National Cancer Institute’s SEER cancer registries and the Centers for Medicare and Medicaid Services Medicare claims data. The SEER program collects cancer incidence and mortality rates from 17 tumor registries across the U.S. covering 28 % of the U.S. population (http://seer.cancer.gov/registries/data.html). The SEER data provides clinical, demographic, and cause of death information for patients diagnosed with cancer. In 2011, Medicare covered 93 % of the U.S. population aged 65 years or older [23]. Medicare claims provide information on covered health care services, including diagnosis and treatment services, which are furnished to Medicare beneficiaries from the time of Medicare eligibility until death.

This study included incident cases of mPC among patients aged 66 years or older diagnosed between 2000 and 2009. The study period ended upon health maintenance organization (HMO) enrollment, Medicare Parts A and B disenrollment, the patient’s death or December 31, 2010 (whichever occurred earlier). Patients were required to have continuous enrollment in Medicare Parts A/B in the year prior to mPC diagnosis in order to assess baseline Charlson comorbidity index (CCI) in the year prior to mPC diagnosis. Patients were excluded from the final sample if they had history of cancer in the five years prior to mPC diagnosis, if their diagnosis month or year was unknown, or if they received a post mortem prostate cancer diagnosis. Patients with an SRE claim prior to mPC diagnosis were also excluded since we were interested only in SREs that were subsequent to mPC diagnosis.

### Measures

#### Step One: SRE prevalence measure

Post-diagnosis SREs were identified using claims that indicated PF, SCC, RAD, or BS. SRE types can be viewed either as clinical events (i.e., PF and SCC) or treatment events that can be administered to prevent a potential event or treat a preceding clinical event (i.e., RAD or BS). Claims involving SREs can be identified from two sets of codes; the International Classification of Diseases 9th version Clinical Modification (ICD-9-CM) and the Healthcare Common Procedure Coding System (HCPCS). The ICD-9-CM is the official system of assigning codes to hospital-related diagnoses and procedures in the US. The HCPCS is a set of health care procedure codes based on the American Medical Association’s Current Procedure Terminology. We identified clinical events (PF and SCC) using ICD-9-CM diagnosis codes and treatment events (RAD and BS) using ICD-9-CM procedure codes and HCPCS codes.

There is some uncertainty associated with identifying SREs from claims because not all SREs can be specifically identified from claims. Therefore, for each SRE, with the exception of BS (since claims for this were clearly indicative of a bone surgical procedure), we created two lists of ICD-9-CM and HCPCS codes that indicated the presence of that SRE subtype. The first list included ICD-9-CM and HCPCS codes that were highly likely to identify SREs (a ‘high specificity’ list), but which could potentially result in underestimating SREs. The second list included additional ICD-9-CM and HCPCS codes that could identify additional SREs but may not necessarily be as specific to the SRE type (a ‘high sensitivity’ list), and therefore had the potential to overestimate SREs. Both lists were reviewed by a medical oncologist and a radiation oncologist until consensus was reached (see Appendix for details). From the ‘high specificity’ and ‘high sensitivity’ lists, we created two SRE definitions. We propose a ‘base case’ SRE definition and provide an ‘alternative’ SRE definition by targeting either sensitivity or specificity, depending on the SRE type.

For PF, the ‘high specificity’ list included only ICD-9-CM Codes (733.1X) that specifically indicated “pathologic fracture” on the claim, whereas the ‘high sensitivity’ list used codes indicating a pathologic fracture or “other fracture” to maximize the identification of all fractures that may have occurred due to cancer. This also accounts for the miscoding potential commonly seen in measuring fractures [24]. However, with the exception of falls on the same level, we excluded “other fracture” if it was associated with accidents or falls within 14 days before the fracture because such fractures are more likely to be associated with the preceding accident or fall instead of weakening of the bone due to cancer. Falls on the same level from routine activities, such as walking or getting out of bed, were included in the study since they are more likely to have been caused by a weakening of the bone due to cancer rather than having been caused by the fall itself. The 14-day window used in the definition of a PF has been used previously by other investigators [11, 13, 14, 17, 19]. The ‘base case’ SRE definition used the PF ‘high sensitivity’ list because medical offices may use “other fracture” codes instead of the more specific “pathologic fracture” codes to indicate a pathologic fracture [24].

For SCC, the ‘high specificity’ list included codes that are commonly and specifically used in the literature to identify SCC, which include unspecified disease of spinal cord ICD-9-CM code 336.9 and HCPCS codes specific to relieving pressure or decompression of the spinal cord. In addition to these codes, the ‘high sensitivity’ list included claims that indicated less specific codes that may or may not indicate an SCC event, such as spondylosis with myelopathy or intervertebral disc disorder with myelopathy. The ‘base case’ SCC definition used the ‘high specificity’ list since many of the procedures in the ‘high sensitivity’ list were not specific to SCC treatment.

For the RAD measure, in Medicare claims there is no indication as to whether radiation is administered to the bone, to the primary cancer site (prostate gland) or to both sites. Therefore, in an attempt to better characterize the RAD measure for the purposes of the present study, we categorized this measure by the *type* of radiation administered, opting for radiation that is more likely to be used for bone palliation compared to local control of disease within the prostate gland. The types of external radiation included in the claims data for men with M1 prostate cancer are: 2-dimensional (2D) and 3-dimensional (3D) radiation, intensity modulated radiation therapy (IMRT), and stereotactic radiosurgery (SRS). Although there may be some outliers, prior to 2009 use of SRS for bone metastasis was negligible, and also likely to be quite low after 2009. Similarly, although IMRT can be used for bone metastasis, its use is still quite uncommon for treating metastatic lesions to the bone even in today’s clinical practice. On the other hand, SRS and IMRT are more likely to be used to treat the prostate than the bone (although this cannot be confirmed from claims data) [25]. Thus, based on such assumptions, our ‘high specificity’ list for radiation to bone included ICD-9-CM and HCPCS codes indicating delivery of 2D or 3D external beam radiation therapy (EBRT) and radioisotope use. The ‘high sensitivity’ list, on the other hand, also includes SRS and IMRT, in addition to 2D/3D radiation and radioisotopes. The ‘base case’ RAD definition used the ‘high specificity’ list of codes. In summary, the “base case” SRE definition included codes identified under the ‘high specificity’ lists of RAD and SCC and codes under the ‘high sensitivity’ list of PF (in addition to all BS codes). The “alternative” SRE definition included codes identified under the ‘high specificity’ list of PF and codes under the ‘high sensitivity’ lists of RAD and SCC (in addition to all BS codes).

#### Step Two: SRE cumulative incidence

Step two requires clustering different SRE claims into SRE episodes to calculate the cumulative incidence. Therefore, a decision had to be made as to which code definition to use for computing the SRE cumulative incidence. We decided to use the base case code definition since it represents the most commonly used approach in the literature. Thus, when counting SREs, we used the 21-day window as the base case since this time period was used in RCTs for measuring SREs. However, this may not be the best approach when using claims since the data capture utilization patterns and it is not clear how to cluster utilization patterns for measuring distinct SREs. Moreover, utilization patterns in the ‘real-world setting’ may differ from the clinical trial setting in that patients are more likely to delay care compared to those enrolled in RCTs. Further, claims are generated for billing purposes and are not designed to reflect the exact dates of service. Given these caveats, we therefore used time periods that were ±7 days from the base case window.

#### Analysis

For step one, we estimated the prevalence of SREs using the two SRE definitions. We examined the frequency distributions between sociodemographic, clinical, and prostate cancer-specific factors using the SRE ‘base case’ and ‘alternative’ SRE definitions. The second step was conditional on using the ‘base case’ definition and involved estimating the cumulative incidence of SREs using three time periods, including 14-, 21-, and 28-day windows. All statistical analyses were performed using SAS software package (version 9.3, SAS Institute, Cary, NC).

## Results

### Characteristics of study sample

The study sample included 8997 mPC patients with a median age of 79 years and observed for a median of 1.5 years (range: 1 day–10 years). Table 1 shows the distribution of other sociodemographic, clinical, and tumor-related characteristics categorized by SRE definition. Among the 8997 mPC patients, 4176 (46 %) experienced an SRE according to the ‘base case’ SRE definition and 3851 (43 %) patients experienced an SRE according to the ‘alternative’ SRE definition. The racial/ethnic composition of the sample was predominantly non-Hispanic White (75 %), with lower proportions of non-Hispanic Blacks (15 %) and Hispanics (6 %).

**Table 1:** Clinical and demographic characteristics of men with mPC according to code definition

			Base case definition					Alternative definition				
	Overall		Yes		No			Yes		No		
	(*N* = 8997)		(*N* = 4176)		(*N* = 4821)			(*N* = 3851)		(*N* = 5146)		
	N	(%)^a^	N	(%)^b^	N	(%)^b^	*P* value	N	(%)^b^	N	(%)^b^	*P* value
Age	1528	17.0	803	52.6	725	47.4	<0.01	794	52.0	734	48.0	<0.01
ᅟ66–70												
ᅟ71–75	1801	20.0	908	50.4	893	49.6		895	49.7	906	50.3	
ᅟ76–80	1995	22.2	942	47.2	1053	52.8		874	43.8	1121	56.2	
ᅟ80<	3673	40.8	1523	41.5	2150	58.5		1288	35.1	2385	64.9	
Race/Ethnicity	6762	75.2	3274	48.4	3488	51.6	<0.01	3001	44.4	3761	55.6	<0.01
ᅟNon-Hispanic White												
ᅟNon-Hispanic Black	1282	14.3	445	34.7	837	65.3		411	32.0	871	68.0	
ᅟHispanic	546	6.1	254	46.5	292	53.5		243	44.5	303	55.5	
ᅟOther	407	4.4	203	49.9	204	50.1		196	48.2	211	51.8	
Married	5216	58.0	2515	48.2	2701	51.8	<0.01	2385	45.7	2831	54.3	<0.01
Charlson Comorbidity Index	4871	54.1	2416	49.60	2455	50.40	<0.01	2280	46.81	2591	53.19	<0.01
ᅟ0												
ᅟ1	1713	19.0	828	48.34	885	51.66		742	43.32	971	56.68	
ᅟ2	777	8.6	333	42.86	444	57.14		289	37.19	488	62.81	
ᅟ3+	722	8.1	286	39.61	436	60.39		259	35.87	463	64.13	
ᅟMissing	914	10.2	313	34.25	601	65.75		281	30.74	633	69.26	
Poor Performance Status	1003	11.2	416	43.21	587	56.79	<0.01	350	37.34	653	62.66	<0.01
Gleason score							<0.01					<0.01
ᅟ2–6	206	2.3	78	37.86	128	62.14		77	37.38	129	62.62	
ᅟ7	663	7.4	287	43.29	376	56.71		285	42.99	378	57.01	
ᅟ8	771	8.6	355	46.04	416	53.96		335	43.45	436	56.55	
ᅟ9	1224	13.6	577	47.14	647	52.86		557	45.51	667	54.49	
ᅟ10	290	3.2	154	53.10	136	46.90		145	50.00	145	50.00	
ᅟNot done/Unknown	5843	64.9	2725	46.64	3118	53.36		2452	41.96	3391	58.04	
Poorly differentiated tumor	3423	38.1	1563	45.66	1860	54.34	0.06	1390	40.61	2033	59.39	<0.01
High PSA at baseline	7298	81.1	3415	46.79	3883	53.21	0.14	3185	43.64	4113	56.36	<0.01

Base case definition included codes identified under the ‘high specificity’ lists of RAD and SCC and codes under the ‘high sensitivity’ list of PF (in addition to all BS codes); Alternative definition included codes identified under the ‘high specificity’ list of PF and codes under the ‘high sensitivity’ lists of RAD and SCC (in addition to all BS codes). The Pearson *χ* ^2^ was used to statistically test for significance of differences in the frequency distributions of categorical variables. A p value of <0.05 was considered statistically significant

^a^column percent

^b^row percent

### SRE prevalence measure

**Fig. 2:**
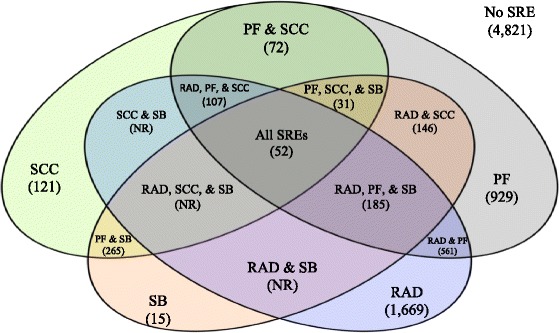
Prevalence of SRE combinations among men diagnosed with mPC from 2000–2009

Among the entire cohort, the proportion of patients with RAD, PF, SCC, and BS were 30, 25, 10, and 8 %, respectively. Of the 4176 patients with an SRE, the proportion of patients with RAD, PF, SCC, and BS were 66, 53, 13, and 14 %, respectively. Table 2 presents how these estimates change if the ‘alternative’ SRE definition was used. Varying the code definition from ‘base case’ to ‘alternative’ resulted in an 8 % increase in the overall SRE prevalence. Sixty nine percent of mPC patients experienced more than one SRE type during the 1.5 year median follow up period (Fig. 2).

**Table 2:** Prevalence of SRE among mPC men according to the ‘base case’ and ‘alternative’ code definitions

	Base case definition			Alternative definition		
	N	% (of full cohort)	% (of patients with at least 1 SRE)	N	% (of full cohort)	% (of patients with at least 1 SRE)
PF	2202	24.5 %	52.7 %	1200	13.3 %	31.2 %
SCC	543	6 %	13 %	738	8.2 %	19.2 %
RAD	2736	30.4 %	65.5 %	2926	32.5 %	76 %
BS^a^	571	6.4 %	13.7 %	571	6.4 %	14.8 %
Any SRE	4176	46.4 %	100 %	3851	42.8 %	100 %

Base case definition included codes identified under the ‘high specificity’ lists of RAD and SCC and codes under the ‘high sensitivity’ list of PF (in addition to all BS codes)

Alternative definition included codes identified under the ‘high specificity’ list of PF and codes under the ‘high sensitivity’ lists of RAD and SCC (in addition to all BS codes)

*SRE* Skeletal Related Event, *PF* Pathological Fracture, *SCC* Spinal Cord Compression, *RAD* Bone Palliative Radiotherapy; *BS* Bone surgery

^a^Only one set of codes was used for bone surgery

### SRE cumulative incidence measure

Cumulative incidence is defined as the number of SREs occurring during the follow up period (median = 1.5 years). Table 3 presents the impact of varying the window length on the SRE cumulative incidence by SRE type. Using the 21-day window, a total of 12,930 SRE episodes were observed during follow up, with RAD, PF, SCC, and BS constituting, respectively, 38, 50, 7, and 5 % of SRE episodes. Varying the window length from 21 days to 28 days resulted in an 8 % decrease in SRE cumulative incidence (RAD: 10 %, PF: 8 %, SCC: 6 %, BS: 0.002 %). Varying the window length from14 days to 28 days resulted in a 20 % decrease in SRE cumulative incidence (RAD: 25 %, PF: 18 %, SCC: 16 %, BS: 0.1 %). The median (mean) number of SREs experienced by mPC patients was 2 (3.1) events during follow up. This was primarily driven by PF since the median (mean) number of PF per patient was 2 (2.9) episodes. Each patient had a mean of 1.1 episodes of BS during follow up.

**Table 3:** Cumulative incidence of SRE types according to window length

	Maximum SRE Episode Length		
	14 days	21 days	28 days
Any Skeletal Related Event			
ᅟCumulative incidence (SRE/patient)	14,869 (3.6)	12,930 (3.1)	11,889 (2.8)
ᅟPatients with # SRE episodes			
ᅟ1	1320 (32)	1557 (37)	1705 (41)
ᅟ2	955 (23)	967 (23)	969 (23)
ᅟ3+	1903 (46)	1652 (40)	1501 (36)
Pathologic Fracture			
ᅟCumulative incidence (PF/patient)	7342 (3.3)	6505 (2.9)	5990 (2.7)
ᅟPatients with # PF episodes (% of SRE Sample)			
ᅟ1	989 (45)	1046 (48)	1097 (50)
ᅟ2	425 (19)	432 (20)	441 (20)
ᅟ3+	794 (36)	724 (33)	662 (30)
Spinal Cord Compression			
ᅟCumulative incidence (SCC/patient)	974 (1.8)	880 (1.6)	823 (1.5)
ᅟPatients with # SCC episodes (% of SRE Sample)			
ᅟ1	377 (70)	401 (74)	423 (78)
ᅟ2	84 (15)	79 (15)	69 (12)
ᅟ3+	82 (15)	63 (11)	51 (10)
Bone Palliative Radiation			
ᅟCumulative incidence (RAD/patient)	5910 (2.2)	4907 (1.8)	4439 (1.6)
ᅟPatients with # RAD episodes (% of SRE Sample)			
ᅟ1	1143 (42)	1470 (54)	1662 (61)
ᅟ2	822 (30)	741 (27)	686 (25)
ᅟ3+	771 (28)	525 (19)	388 (14)
Surgery to Bone			
ᅟCumulative incidence (BS/patient)	643 (1.1)	638 (1.1)	637 (1.1)
ᅟPatients with # BS episodes (% of SRE Sample)			
ᅟ1	510 (89)	514 (90)	515 (90)
ᅟ2+	61 (11)	57 (10)	56 (10)

*SRE* Skeletal related event, *PF* Pathological fracture, *SCC* Spinal cord compression, *RAD* Bone palliative radiotherapy; *BS* Bone surgery

The median (mean) time from mPC diagnosis to the first SRE was 154 (335) days using the ‘base case’ definition and 133 (303) days using the ‘alternative’ definition. In addition, the median (mean) time from the first SRE to the second SRE was 21 (177) days using the ‘base case’ definition and 26 (192) days using the ‘alternative’ definition. The median (mean) time from mPC diagnosis to death was 566 (729) days using the ‘base case’ SRE definition and 568 (734) days using the ‘alternative’ SRE definition.

## Discussion

The goal of the study was to quantify the differences in prevalence and cumulative incidence of SREs based on different measurement approaches for a clinically well-defined population of men using health care claims data. Our findings suggest that SRE prevalence and cumulative incidence obtained from administrative claims data can be affected by the methods used to estimate these measures. For example, the prevalence of SREs varied depending upon the types of ICD-9-CM and HCPCS codes used to identify SREs from claims. While all SRE subtypes were impacted by the codes used, PF was the driver of the variability in the overall SRE prevalence. Our findings also suggest that the window length used to cluster SRE claims into a single SRE affects the cumulative incidence of individual SREs, particularly RAD. This may probably be the case because RAD is given in cycles lasting generally for two to four weeks. Therefore, it is not uncommon to observe multiple RAD claims in a one-month period making the RAD measure highly sensitive to the window length.

Given that metastasis to the bone from various cancers is a major contributor to patient morbidity and mortality, significant efforts have been made to develop therapies to mitigate and alleviate complications of bone metastasis, known collectively as SREs. Significant progress has been made over the years with bone targeted therapies, as determined by RCTs, which has led to the widespread use of such therapies in advanced cancers. To better understand the impact of these treatments when applied outside the context of clinical trials, where by necessity the experience is confined to limited numbers of patients who are rigorously selected, observational studies have proven invaluable. Despite some of the inherent limitations associated with observational studies, they provide important information when therapies developed via RCTs are taken to the real world setting and can help inform healthcare decisions and policy. Therefore, using mPC as a model, we undertook the present study to determine whether and how SRE-related data may be affected by certain parameters, such as codes and window lengths, that define the boundaries of the SRE-related observational datasets.

Investigators have used different approaches to identify SREs from claims. Figure 1 illustrates the various approaches in the literature used to identify SREs from claims. For PF, two approaches have been used. The more specific approach was to use codes indicating ‘pathological fracture’ [15], while the second and more sensitive approach was to use codes indicating ‘pathological fracture’ or ‘other fracture’ (if there were no concurrent claims with codes suggesting trauma or accident, excluding accident codes for routine activity ‘falls on the same level’) [11, 13, 14, 17, 19]. When identifying SCC, some investigators exclusively used ‘unspecified disease of spinal cord’ diagnosis code [14, 19]. Others have included additional ICD-9-CM diagnosis codes such as ‘spondylogenic compression’ and ‘intervertebral disc disorder with myelopathy’ and/or procedure codes indicating ‘laminoplasty’ or ‘decompression of spinal cord.’ [11, 15] For RAD, investigators who aimed for high sensitivity included any claim indicating receipt of radiation or a radiation-related procedure (examples include ‘medical radiation physics consultation,’ ‘computed tomography guidance for placement,’ and ‘radiation calculations’) [12]. On the other hand, investigators aiming for higher specificity only used claims indicating radiation delivery in the form of EBRT [17], sometimes with radiopharmaceuticals [14]. Some investigators required receipt of RAD from a ‘radiation oncologist’ or a ‘therapeutic radiologist’ [13]. Other investigators considered RAD only when the claim was concurrent with a bone metastasis, PF, SCC, or bone pain [11]. Similar to PF, some investigators required that BS be identified as an SRE if it was not preceded by an accident or trauma in the 14 days prior to the surgery [11]. Additionally, a few investigators have addressed the issue of how to handle multiple types of SRE claims that occur on the same day by imposing a hierarchy in which SREs viewed as clinical events (i.e., PF and SCC) take precedence over SREs viewed as treatment events (i.e., RAD and BS). For example, RAD or BS would only be counted if there were no claims for PF or SCC on the same day [11, 15]. Given the great degree of variation in the approaches to identify different SRE types, this study investigates the consequences of various approaches and quantifies the variation in prevalence and cumulative incidence measures that can occur depending upon the approach used to study such events.

This study has several limitations. The algorithms used to identify SREs in this study have not been validated and are subject to further research. In another setting, A Danish study validated the ICD-10 coding of bone metastasis and SREs in 100 prostate cancer patients in the National Registry of Patients against medical chart review data and found that the sensitivity of ICD-10 codes ranged from 44–55 % and specificity ranged from 94–100 %. The authors view these numbers to represent sufficient sensitivity and high specificity. [26] Identifying SREs from claims is not a straightforward task because there is no billing code for SREs. The RAD measure represents a particular challenge for estimating the true prevalence of RAD from claims data. This is evident by the variability in the estimates using different claims and is an important finding of this study. We believe the main reason for that variability is that the RAD measure may identify receipt of radiation as local treatment of the prostate instead of the bone. We have attempted to minimize the potential for overestimating the prevalence by only assessing patients whose cancer has metastasized and are therefore, more likely to have radiation administered to metastatic sites. For patients with incident mPC, the initial treatment tends to be androgen deprivation therapy, with radiation to the primary site being less likely (unless significant local complications such as obstructive uropathy, hematuria or locoregional pain occur from the cancer within the prostate gland during the course of the disease). Thus, the RAD measure in Medicare claims among mPC patients is more likely to reflect radiation therapy to treat bone metastasis related issues. We also minimized the potential for overestimation by identifying radiation techniques more likely to be administered to the bone (EBRT and radiopharmaceuticals) as opposed to the prostate gland consistent with the literature [14, 17]. Among patients with mPC, a small proportion may have oligometastatic prostate cancer and may receive radiation to the prostate with a curative intent [27]. However, these patients constitute very small numbers and their inclusion in the study sample is not likely to impact the findings. Nevertheless, the lack of radiation site specifications in the claims data represents some of the limitations inherent in the RAD measure when using claims.

The PF measure can also pose challenges. While there are specific ICD-9 diagnosis codes that indicate “pathologic fracture” and others that specifically indicate “other fractures” in the ‘musculoskeletal and connective tissue chapter’ of the ICD-9-CM handbook, there is confusion as to which set of codes to include when defining cancer-related fractures and osteoporosis-related fractures. The definition of “pathologic fracture” according to the American Hospital Association coding manual used by physicians in the U.S. is a “break in a diseased bone due to weakening of bone structure by pathologic processes without identifiable trauma” (http://www.eicd.com/guidelines/default.htm). However, Curtis et al recommend including pathologic fractures in the definition of osteoporosis-related fractures because physicians can interpret one of the pathologic processes to be osteoporosis [24]. Similarly, there is a potential for oncologists to miscode pathologic fractures due to cancer as “other fractures.” Therefore, for PF a more sensitive measure may be more appropriate when using claims. For SCC, the use of all procedure codes that involve decompression of the spinal cord, as well as the more specific diagnosis codes, may be more accurate since many of the previously used diagnosis codes were not specific to SREs. Additionally, this study did not include younger patients (<66 years) diagnosed with incident mPC or elderly patients who were initially diagnosed with non-metastatic disease but developed bone metastasis during follow up. Moreover, the study population included patients with M1 disease, not specifically M1b disease making the denominator larger and potentially underestimating the true prevalence and cumulative incidence of SREs.

## Conclusions

Using different claims-based approaches to study the burden of SREs can yield different estimates. We found that SRE prevalence was affected by the codes used, with PF being most impacted. The overall SRE cumulative incidence was affected by the window length used, with RAD being most affected. These results underscore the importance of the baseline definitions used to study claims data when attempting to understand relevant clinical events such as SREs in the real world setting. Over the past 30 years, both RCTs and observational studies have played critical roles in informing treatment decision making for patients with mPC. Measurement approaches employed in RCT may not translate readily to the observational study setting when using health care claims data. In addition, the patterns of utilization associated with clinically diagnosed conditions may differ between the trial and observational study setting, further complicating the task of identifying SREs based on utilization data using approaches employed in RCTs. Anchoring on the approach used in the trial settings, we provide a proof of concept investigation of the implications of different measurement approaches for prevalence and cumulative incidence estimates in a clinically well-defined population of older men. Building on these findings to develop validated algorithms for use in the observational study setting will bring needed consistency to the measurement of SREs using administrative claims data. A consistent approach for assessing SREs in administrative claims will not only improve comparability across studies but also has the potential to address questions relevant to comparative effectiveness research in the area of bone metastasis.

## Appendix

**Table 4:** ICD-9 and HCPCS codes used for base case (**bolded**) and alternative SRE prevalence measures

**SPINAL CORD COMPRESSION**	
*ICD-9*	**3369**, 7211, 7214, 72141, 72142, 72191, 7227, 72270, 72271 and 72273
*HCPCS*	**63050, 63051, 22551, 22552, 63064, 63066, 61343, s2348, 63075-8, s2350, s2351, 63195, 63197, 63199, 63001, 63003, 63005, 63011, 63015, 63016, 63017, 63170, 63012, 63045, 63046, 63047, 63048, 63040, 63042, 63043, 63044, 63020, 63030, 63035, 22224, 22222, 22214, 22212, 22207, 22206, 0274t, 0275t, c9729, 0202t, 22865, 0164t, 0094t, 0097t, 63057, 63056, 63055, 63081, 63082, 63087, 63088, 63101, 63102, 63103, 63090, 63091, 63086, and 63085**
**PATHOLOGIC FRACTURES**	
*ICD-9*	**7331, 73311, 73312, 73313, 73314, 73315, 73316, and 73319**
*HCPCS*	8202, 8208, 8210, 8212, 73311, 8120, 8122, 8124, 73312, 8130, 8132, 8134, 8138, 73316, 8230, 8232, 8238, 73313, 805, 806, 8200, 7331, 73310, 73319, 800, 807, 8080, 8082, 8084, 8088, 8100, 8240, 8242, 80701, 80702, 80703, 80704, 80705, 80706, 80707, 80708, 80709, 80841, 80842, 80843, and 80849
**TRAUMA/NON-ROUTINE FALLS / ACCIDENTS**	
*ICD-9*	819, 828, 851, 852, 853, 854, 860, 861, 862, 863, 864, 865, 866, 867, 868, 869, 8074, 9584, 80712, 80713, 80714, 80715, 80716, 80717, 80718, 80719, E800-E848, E881, E882, E883, E884.0, E884.1, E884.5, E885.0, E885.1, E885.2, E885.3, E885.4, E886.0, E886.9, E888.0, and E888.1
**BONE PALLIATIVE RADIOTHERAPY**	
*ICD-9*	**9223, 9224, 9229**, 9230, 9231, 9232, and 9239
*HCPCS*	**A9600, A9604, A9605, C9401,** G0173, G0174, G0243, G0251, G0339, G0340, **J3005**, 0073 T, 61793, 61796, 61797, 61798, 63620, 63621,77371, 77372, 77373, **77401, 77402, 77403, 77404, 77406, 77407, 77408, 77409, 77411, 77412, 77413, 77414, 77416, 77418, 79005, 79101, 79200, 79300, 79400, 79403, 79440, 79445, and 79999**
**BONE SURGERY**	
*ICD-9*	**7815, 7845, 7855, 7915, 7925, 7935, 7995, 7812, 7842, 7852, 7911, 7921, 7931, 7991, 7813, 7843, 7853, 7912, 7922, 7932, 7992, 7817, 7847, 7857, 7916, 7926, 7936, 7996, 0353, 8102, 8103, 8104, 8105, 8106, 8107, 8108, 7810, 7811, 7816, 7819, 7840, 7841, 7846, 7849, 7850, 7851, 7856, 7859, 7910, 7919, 7920, 7929, 7930, 7939, 7990, and 7999**
*HCPCS*	**27187, 27235, 27236, 27244, 27245, 27248, 27269, 27495, 27506, 27507, 27509, 27511, 27513, 27514, 23615, 23616, 23630, 24498, 24515, 24516, 24538, 24545, 24546, 24566, 24575, 24579, 24582, 24586, 24587, 24635, 24665, 24666, 24685, 25490, 25491, 25492, 25515, 25525, 25526, 25545, 25606, 25607, 25608, 25609, 27535, 27536, 27745, 27756, 27758, 27759, 27766, 27769, 27784, 27792, 27826, 27827, 22325, 22326, 22327, 22328, 22520, 22521, 22522, 22532, 22533, 22534, 22548, 22550, 22554, 22555, 22556, 22558, 22565, 22585, 22590, 22595, 22600, 22610, 22612, 22614, 22615, 22625, 22630, 22632, 20982, 23490, 23515, 23585, 27215, 27216, 27217, 27218, 27226, 27227, 27228, 27524, 27540, 22523, 22524, 22525, 22526, 22527, 25574, and 25575**

## Abbreviations

2D: 2-dimensional
3D: 3-dimensional
BS: Bone surgery
CCI: Charlson comorbidity index
EBRT: External beam radiation therapy
FDA: Food and Drug Administration
HCPCS: Healthcare Common Procedure Coding System
HMO: Health maintenance organization
ICD-9-CM: International Classification of Diseases 9^th^ version Clinical Modification
IMRT: Intensity modulated radiation therapy
mPC: Metastatic prostate cancer
PF: Pathologic fracture
RAD: Bone palliative radiotherapy
RCT: Randomized clinical trial
SCC: Spinal cord compression
SEER: Surveillance, Epidemiology, and End Results
SRE: Skeletal related event
SRS: Stereotactic radiosurgery
